# Effects of Constitutive and Acute Connexin 36 Deficiency on Brain-Wide Susceptibility to PTZ-Induced Neuronal Hyperactivity

**DOI:** 10.3389/fnmol.2020.587978

**Published:** 2021-01-11

**Authors:** Alyssa A. Brunal, Kareem C. Clark, Manxiu Ma, Ian G. Woods, Y. Albert Pan

**Affiliations:** ^1^Center for Neurobiology Research, Fralin Biomedical Research Institute at Virginia Tech Carilion, Virginia Tech, Roanoke, VA, United States; ^2^Translational Biology Medicine and Health Graduate Program, Virginia Tech, Blacksburg, VA, United States; ^3^Department of Biology, Ithaca College, Ithaca, NY, United States; ^4^Department of Biomedical Sciences and Pathobiology, Virginia-Maryland College of Veterinary Medicine, Virginia Tech, Blacksburg, VA, United States; ^5^Department of Psychiatry and Behavioral Medicine, Virginia Tech Carilion School of Medicine, Roanoke, VA, United States

**Keywords:** MAP-mapping, epilepsy, seizure, gap junction (connexin), brain mapping

## Abstract

Connexins are transmembrane proteins that form hemichannels allowing the exchange of molecules between the extracellular space and the cell interior. Two hemichannels from adjacent cells dock and form a continuous gap junction pore, thereby permitting direct intercellular communication. Connexin 36 (Cx36), expressed primarily in neurons, is involved in the synchronous activity of neurons and may play a role in aberrant synchronous firing, as seen in seizures. To understand the reciprocal interactions between Cx36 and seizure-like neural activity, we examined three questions: (a) does Cx36 deficiency affect seizure susceptibility, (b) does seizure-like activity affect Cx36 expression patterns, and (c) does acute blockade of Cx36 conductance increase seizure susceptibility. We utilize the zebrafish pentylenetetrazol [PTZ; a GABA(A) receptor antagonist] induced seizure model, taking advantage of the compact size and optical translucency of the larval zebrafish brain to assess how PTZ affects brain-wide neuronal activity and Cx36 protein expression. We exposed wild-type and genetic Cx36-deficient (*cx35.5-/-*) zebrafish larvae to PTZ and subsequently mapped neuronal activity across the whole brain, using phosphorylated extracellular-signal-regulated kinase (pERK) as a proxy for neuronal activity. We found that *cx35.5-/-* fish exhibited region-specific susceptibility and resistance to PTZ-induced hyperactivity compared to wild-type controls, suggesting that genetic Cx36 deficiency may affect seizure susceptibility in a region-specific manner. Regions that showed increased PTZ sensitivity include the dorsal telencephalon, which is implicated in human epilepsy, and the lateral hypothalamus, which has been underexplored. We also found that PTZ-induced neuronal hyperactivity resulted in a rapid reduction of Cx36 protein levels within 30 min. This Cx36 reduction persists after 1-h of recovery but recovered after 3–6 h. This acute downregulation of Cx36 by PTZ is likely maladaptive, as acute pharmacological blockade of Cx36 by mefloquine results in increased susceptibility to PTZ-induced neuronal hyperactivity. Together, these results demonstrate a reciprocal relationship between Cx36 and seizure-associated neuronal hyperactivity: Cx36 deficiency contributes region-specific susceptibility to neuronal hyperactivity, while neuronal hyperactivity-induced downregulation of Cx36 may increase the risk of future epileptic events.

## Introduction

Connexins are transmembrane proteins that oligomerize to form a transmembrane pore called a hemichannel, which enables the exchange of molecules between the extracellular space and cell interior. Two hemichannels between adjacent cells can dock and form a continuous pore, known as a gap junction, allowing for direct intercellular coupling.

Several types of connexin proteins are expressed within the brain, both in non-neuronal cells and neurons. Connexin 43 (Cx43) is expressed in astrocytes and plays an important role in ion homeostasis at the synapse (Ca^2+^ and K^+^) (Cotrina et al., 1998; Kofuji and Newman, 2004). Through an interconnected network of Cx43-coupled astrocytes, ions can be shunted away from synapses and certain areas of the brain (Kofuji and Newman, 2004). The dysfunction of this process may lead to the malfunction of neural networks. As such, Cx43 has been shown to play an important role in the development of epilepsy (Vincze et al., 2019). Connexin 36 (Cx36) is the main connexin expressed by neurons and forms inter-neuronal gap junctions (electrical synapses), which are responsible for fast synaptic transmission and the synchronous firing of neurons within the brain (Rash et al., 2012). It is involved in brain functions that rely on synchronous firing such as learning and memory (Allen et al., 2011; Wang and Belousov, 2011), retina visual processing (Kovács-Öller et al., 2017), and sensorimotor reflex in the zebrafish (Miller et al., 2017). As the key structural component of electrical synapses, Cx36 may also act as a therapeutic target in diseases involving deficiencies in fast communication and aberrant synchronous firing, such as seizures. However, the reciprocal relationships between the Cx36 and seizures have remained unclear.

Previous work has examined the roles of Cx36 in the pathogenesis of seizures, but there has been no consensus on whether Cx36 increases or decreases seizure susceptibility (Gajda et al., 2005; Jacobson et al., 2010; Voss et al., 2010; Shin, 2013). Jacobson et al. (2010) found that Cx36 knockout mice exhibited an increase in seizure-like behaviors following the administration pentylenetetrazol (PTZ; a GABA(A)-receptor antagonist), indicating that normal expression of Cx36 may be protective against seizure-inducing conditions. However, this finding contradicts studies using the connexin blocking drug quinine, which found the drug either decreased the severity of seizures (Gajda et al., 2005) or showed no change (Voss et al., 2010). The discrepancy may potentially be due to the difference between chronic Cx36 deficiency (Cx36 knockout) vs. acute Cx36 deficiency (quinine). However, quinine has broad antagonistic activity against many different connexins expressed in the nervous system, and the effects cannot be attributed solely to the inhibition of Cx36 (Cruikshank et al., 2004; Manjarrez-Marmolejo and Franco-Pérez, 2016). Additionally, the difference in seizure induction methods and seizure metrics also makes direct comparisons between studies problematic.

Previous findings are also mixed regarding how neuronal hyperactivity affects the expression of Cx36. In rodent seizure models and epilepsy patient post-mortem samples, some groups have found that Cx36 expression was increased (Collignon et al., 2006; Laura et al., 2015; Wu et al., 2017), while others found decreased Cx36 expression (Söhl et al., 2000; Condorelli et al., 2003) or no change (Motaghi et al., 2017). Furthermore, even though seizures result in brain-wide changes in neuronal connectivity (Morgan et al., 2010), seizure-induced changes in Cx36 expression had only been examined in the dorsal telencephalon (cortex and hippocampus) (Condorelli et al., 2003; Laura et al., 2015; Motaghi et al., 2017; Wu et al., 2018). Potential changes to Cx36 expression in other brain areas following neuronal hyperactivity remain unknown.

To further investigate the relationship between Cx36 and neuronal hyperactivity and address the technical limitations listed above, we employ zebrafish as an experimental system. The small size of zebrafish larvae facilitates imaging of the whole brain under a laser scanning confocal microscope, which provides a unique opportunity to examine whole-brain activity as well as Cx36 protein regulation in an intact vertebrate organism. Additionally, the PTZ-induced seizure model in zebrafish has been well-characterized physiologically and behaviorally and is an effective model in identifying therapeutics to target epilepsy in humans (Baxendale et al., 2012; Baraban et al., 2013). Previous studies using rodents and zebrafish have examined the diverse effects of PTZ in different brain regions (Shehab et al., 1992; Nehlig, 1998; Baraban et al., 2005; Szyndler et al., 2009; Baxendale et al., 2012; Diaz Verdugo et al., 2019; Liu and Baraban, 2019; Yang et al., 2019). These region-specific effects can be effectively captured in the zebrafish using the recently developed MAP-map technique utilizing phosphorylated extracellular-signal-regulated kinase (pERK) as a proxy for neuronal activity (Randlett et al., 2015).

In zebrafish, Cx36 proteins are encoded by four genes: *cx35.1, cx34.7, cx34.1*, and *cx35.5* (Miller et al., 2017). In culture, all four isoforms are recognized by an anti-human Cx36 antibody, and the loss of either *cx34.1* or *cx35.5* resulted in the greatest reduction in brain-wide Cx36 antibody labeling. In contrast, loss of *cx34.7* and *cx35.1* have minimal effects on global Cx36 levels. Interestingly, the expression of Cx34.1 and Cx35.5 are mutually dependent. For example, in the *cx35.5* loss-of-function animals (*cx35.5-/-*), the majority of anti-Cx36 labeling is lost, with weak residual labeling from Cx34.1(Miller et al., 2017).

Using zebrafish, we created a whole-brain activity map following hyperactivity using the MAP-mapping method (Randlett et al., 2015) to determine that there are both dose-varying and region-specific changes in neuronal hyperactivity following administration of PTZ. Additionally, we created a whole-brain expression map of Cx36 following the administration of PTZ. With this, we determined specific brain regions that showed decreases in Cx36 expression following hyperactivity. Finally, by acutely reducing the function of Cx36 using the Cx36 blocking drug, mefloquine, we determined that acute inhibition of Cx36 is detrimental, and leaves the animal more susceptible to PTZ-induced hyperactivity than their untreated counterparts.

## Methods

### Zebrafish Husbandry

All zebrafish used in this study were pigmentless (*nacre-/-*) in a mixed background of AB and TL wild-type strains (Zebrafish International Resource Center). *cx35.5* (ZFIN gene symbol: *gjd2a*) heterozygotes were gifts from Dr. Adam Miller at the University of Oregon. The *cx35.5* mutant was generated by TALEN-mediated genome targeting, which generated a frameshift mutation (5 bp deletion in exon 1) and polypeptide truncation (Shah et al., 2015). Zebrafish embryos and larvae were raised under 14 h light/10 h dark cycle at 28.5°C in water containing 0.1% Methylene Blue hydrate (Sigma-Aldrich). Sex is not a relevant variable for the larval stages being used (0–6 days post-fertilization, dpf), as laboratory zebrafish remain sexually undifferentiated until 2 weeks of age (Maack and Segner, 2003; Wilson et al., 2014). All husbandry procedures and experiments were performed according to protocols approved by the Institutional Animal Care and Use Committee at Virginia Tech.

### Immunohistochemistry

Zebrafish larvae were fixed overnight in 4% paraformaldehyde (PFA) on a rocker at 4°C. Samples were then processed and stained as previously described by Randlett et al. (2015). Primary antibodies that were used are as follows: p44/42 MAPK (tERK) (4696S, Cell Signaling Technologies), Phospho-p44/42 MAPK (pERK) (4370S, Cell Signaling Technologies), and Anti-activated caspase-3 (559565, BD Pharmingen). For the anti-Connexin36 antibody (36-4600, Invitrogen), fish were fixed in 2% trichloroacetic acid (TCA) for 3 h, and sample processing and staining were performed as previously described (Marsh et al., 2017).

### Brain Activity Mapping (MAP-mapping)

6 dpf wild-type and *cx35.5* mutant zebrafish larvae were first acclimated for 15 min in a 6-well plate and then transferred into a well-containing 0 mM (E3 embryo media only), 2, 5, 10, or 20 mM PTZ in embryo media for 15 min. Larvae were then fixed in 4% PFA overnight and immunostained and imaged using a Nikon A1 confocal microscope. Subsequent MAP-mapping analyses were performed as previously described (Randlett et al., 2015). Statistical significance was determined through the Mann-Whitney *U* statistic, calculating statistically significant changes in the pERK/tERK ratio for each registered voxel across all samples within a group. Multiple comparison correction was done using a false discovery rate (FDR)-based method, with FDR threshold set at 0.005%. The FDR threshold was calculated for each MAP-map comparison by randomizing pixel data into pseudogroups over 500 iterations. The pixels with Z-score (calculated from the Mann-Whitney *U* statistic) above the FDR threshold are considered significant. For pixels with significant Z-score, they are assigned an intensity value based on delta-median (0–0.5 delta-median maps to 0–65535). The mean intensity value for each region was calculated by dividing the total pixel intensity value by the total area. The positive (more active) and negative (less active) intensity values were calculated separately (i.e., they do not cancel each other out.) Regions with values greater or lesser than zero were considered significantly different in the given comparison. Brain regions highlighted in the text of this document were selected based on the following criteria: only brain regions were selected (individual neuron clusters were not mentioned), and only brain regions with well-defined functions were selected to be highlighted. All identified brain regions and neuron clusters can be found in the Supplementary Tables.

### Cx36 Expression Map

At 6 dpf, larvae were acclimated for 15 min in a 6-well plate with embryo media and then transferred into a well containing 20 mM PTZ for either 30 min or 1 h. Larvae were then either fixed immediately or allowed to recover for 1, 3, 6, or 24 h in embryo media. Larvae were fixed in 2% trichloroacetic acid (TCA) for 3 h and immunostained with antibodies against Connexin 36 and tERK (Miller et al., 2017). Confocal images were then morphed to a tERK standard brain image stack using CMTK (Randlett et al., 2015). To subtract background signal, an average stack of *cx35.5-/-* fish morphed and stained in the same way was subtracted from all images and then were processed as previously described for MAP-mapping, except for replacing pERK with the morphed and background subtracted anti-Cx36 (Randlett et al., 2015).

### Cell Death Quantification

At 6 dpf, mutant and wild-type larvae were first acclimated for 15 min in a 6-well plate and then transferred into a well-containing either embryo medium or 20 mM PTZ for 1 h. Larvae were then immediately fixed in 4% PFA overnight, and immunostained. Images were morphed to a standard brain and analyzed as previously described (Randlett et al., 2015). ROIs for the diencephalon, mesencephalon, telencephalon from ZBrain were then overlaid on each stack, and Caspase positive cells were counted in each ROI. Standard unpaired *t*-tests with Holm-Sidak's correction for multiple comparisons were run between each group in GraphPad Prism.

### Mefloquine Treatment

At 6 dpf, larvae were exposed to either 0.025% DMSO (vehicle group) or 25 μM mefloquine. After 3 h of exposure, fish and their relative media (either DMSO or mefloquine) were transferred to a 6-well plate and allowed to acclimate for 15 min. Larvae were then transferred to embryo media with 0, 2, 5, 10, or 20 mM PTZ for 15 min. Larvae were then immediately fixed in 4% PFA overnight, immunostained, and imaged using a Nikon A1 confocal microscope. Subsequent analysis was performed as previously described (Randlett et al., 2015).

### Image Processing and Statistical Analysis

Images were processed and quantified using Fiji (Schindelin et al., 2012). MATLAB 2019 (MathWorks) was used for MAP-mapping analysis (Randlett et al., 2015). For Caspase-3 quantification, statistical analyses were performed in GraphPad Prism (Version 8). An unpaired *t*-test with Holm-Sidak's correction for multiple comparisons was performed. Results were considered significant if *p* < 0.05. Raw data will be available upon reasonable request.

## Results

### PTZ Induces Brain-Wide Neuronal Hyperactivation in a Dose-Dependent Manner

**Figure 1:**
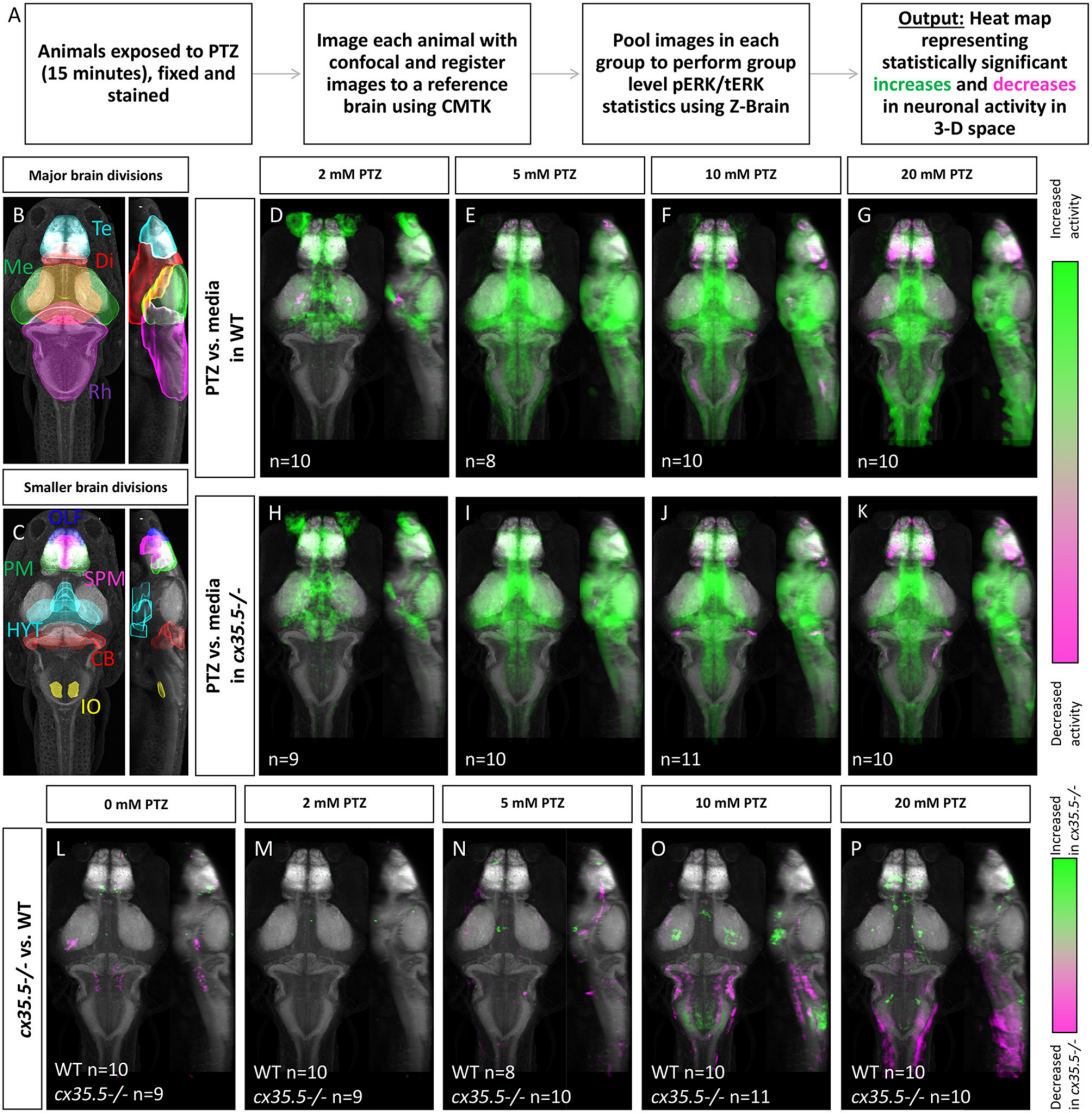
Whole-brain activity map showing significant regional differences in neuronal activity following various PTZ concentration exposure in wild-type and *cx35.5-/-* zebrafish larvae. **(A)** Schematic describing the data collection and analysis process for whole-brain activity mapping using the MAP-mapping technique. **(B)** Representative atlas depicting dorsal and lateral views of the 4 major brain divisions in zebrafish, showing the telencephalon (Te) in cyan, the diencephalon (Di) in red, the mesencephalon (Me) in green, and the rhombencephalon (Rh) in magenta. **(C)** Representative atlas depicting smaller brain regions mentioned in the text. The olfactory bulb (OLF) is labeled in blue, the pallium (PM) in green, the subpallium (SPM) in magenta, the hypothalamus (rostral, intermediate, and caudal) in cyan (HYT), the cerebellum (CB) in red, and the inferior olive (IO) in yellow. **(D–G)** Colors indicate ROIs with increased (green) or decreased (magenta) pERK/tERK ratio in wild-type PTZ treated groups compared to wild-type media only group. **(D)** 2 mM PTZ treated (*n* = 10) **(E)** 5 mM PTZ treated (*n* = 8) **(F)** 10 mM PTZ treated (*n* = 10) and **(G)** 20 mM PTZ treated (*n* = 10) vs. media (*n* = 10). **(H–K)** Colors indicate ROIs with increased (green) or decreased (magenta) pERK/tERK ratio in *cx35.5-/-* larvae PTZ treated groups compared to *cx35.5*-/- media only group. **(H)** 2 mM PTZ treated (*n* = 9) **(I)** 5 mM PTZ treated (*n* = 10) **(J)** 10 mM PTZ treated (*n* = 11) and **(K)** 20 mM PTZ treated (*n* = 10) vs. media (*n* = 9). **(L–P)** Colors indicate ROIs with increased (green) or decreased (magenta) pERK/tERK ratio in *cx35.5-/-* groups compared to corresponding wild-type groups. **(L)** Media treated (WT *n* = 10, MUT *n* = 9) **(M)** 2 mM PTZ treated (WT *n* = 10, MUT *n* = 9) **(N)** 5 mM PTZ treated (WT *n* = 8, MUT = 10) **(O)** 10 mM PTZ treated (WT *n* = 10, MUT *n* = 11) and **(P)** 20 mM PTZ treated (WT *n* = 10, MUT *n* = 10).

PTZ inhibits GABA(A) receptor-mediated inhibitory neurotransmission, which leads to global neuronal hyperactivation and seizure-like neurological and behavioral phenotypes in both rodents and zebrafish (Baraban et al., 2005). To determine whether different brain regions have distinct sensitivities to PTZ-induced neuronal hyperactivation, we compared whole-brain activity in wild-type fish exposed to varying concentrations of PTZ. To do this, we utilized the MAP-mapping assay to create whole-brain activity maps (Randlett et al., 2015) (Figure 1A). MAP-mapping offers a snapshot of neuronal activity by utilizing the ratio of total extracellular signal-regulated kinase (tERK), which is present in all neurons, and phosphorylated ERK (pERK), the phosphorylated form of ERK that is induced within 5 min (Ji et al., 1999; Dai et al., 2002; Cancedda et al., 2003) following neuronal activity. The ratiometric pERK/tERK signal can then be quantified for individual, registered brain image stacks and statistically tested in an annotated 3D brain atlas, Z-Brain (Randlett et al., 2015). Statistical significance was determined through the Mann-Whitney *U* statistic, calculating statistically significant changes in the pERK/tERK ratio for each registered voxel across all samples within a group. Voxels that show statistically significant increases in pERK/tERK ratio are shown in green and statistically significant decreases in pERK/tERK ratio are shown in magenta. By averaging pERK across a large group of animals (7–20), MAP-map generates a spatially precise snapshot of average neuronal activity in a given group. This method allows us to assess the effects of PTZ on average neuronal activity, though the timing and location of ictal events cannot be determined due to the long temporal integration of ERK signaling.

Using MAP-mapping, we found region-specific changes in neuronal activity in response to varying concentrations of PTZ. We treated wild-type animals by bath-exposing them to embryo media with 2, 5, 10, and 20 mM PTZ for 15 min. Animals exposed to media only were used as the baseline for comparison. Neuronal activity was measured by the pERK/tERK ratio as described previously (Randlett et al., 2015). For a complete list of statistically significant changes by brain region in neuronal activity, see Supplementary Table 2. After exposure to 2 mM PTZ, we saw moderate increases in neuronal activity in more restricted brain areas in regions responsible for homeostatic regulation (hypothalamus and preoptic area) and executive functioning (subpallium, pallium) as well as the cerebellum (Figure 1D), see Figure 1 for an atlas of zebrafish brain regions. After exposure to 5, 10, and 20 mM PTZ, we observed broader increases in brain-wide neuronal activity (Figures 1E–G). These regions include those that were activated by 2 mM PTZ (hypothalamus, preoptic area, subpallium and in many regions involved in movement control such as the pretectum, cerebellum, and oculomotor nuclei. Additionally, we observed some brain areas that became less active after exposure to PTZ: the telencephalon was less active at 10 and 20 mM PTZ than at lower concentrations (Figure 1G) and the olfactory bulb was less active across all PTZ concentrations (Figures 1D–G). The complete list of all identified changes is provided in Supplementary Table 1.

Overall, we were able to generate a PTZ dose-varying whole-brain activity map in 6 dpf zebrafish. We saw increased neuronal activity in areas previously identified to be involved in PTZ induced hyperactivity such as the pallium and optic tectum (Liu and Baraban, 2019). We also identified additional regions that were previously unidentified such as the hypothalamus.

### Genetic *cx35.5* Deficiency Results in Changes in PTZ-Induced Brain-Wide Neuronal Hyperactivity

To understand what effect loss of Cx36 has on hyperactivity we examined whole-brain activity changes at different concentrations of PTZ in the *cx35.5-/-* larvae. *cx35.5-/-* fish have a complete loss of the Cx35.5 isoform of Cx36 as well as a significant reduction in the other three Cx36 isoforms (Miller et al., 2017 and also **Figures 3A,B**). However, it is important to note some residual expression from non-Cx35.5 isoforms does remain and the mutant is, therefore, not a full knock-out of all Cx36 isoforms (Miller et al., 2017). We again employed the MAP-mapping technique to determine which brain regions show a significant difference between PTZ-treated mutants and untreated mutants. Similar to their wild-type siblings, at 2 mM PTZ, significant increases in neuronal activity were seen in the diencephalon (preoptic area and hypothalamus) and telencephalon (subpallium), were observed (Figure 1H) see Figure 1 for an atlas of zebrafish brain regions. Additionally, we saw increases in the diencephalon (retinal arborization fields) associated with visual processing (Figure 1H). At 5, 10, and 20 mM PTZ, we found a very similar map to that of their wild-type siblings, with increases and decreases in many of the same major brain regions listed previously (Figures 1E–G,I–K). For a complete list of significantly change brain regions, see Supplementary Table 1.

### Changes in *cx35.5-/-* Whole-Brain Activity Maps Compared to Wild Type

To understand differences in neuronal hyperactivity between *cx35.5-/-* and wild-type animals, we compared the activity map of *cx35.5-/-* and wild-type siblings at baseline (media only) and after exposure to different concentrations of PTZ (Figures 1L–P). We observed no increases in neuronal activity at baseline, however, we did observe decreases in activity in *cx35.5-/-* relative to wild-type in the rhombencephalon reticulospinal neurons and medial vestibular neurons (Figure 1L), see Figure 1 for an atlas of zebrafish brain regions. At 2 mM PTZ, there were no significant changes in brain-wide neuronal activity between *cx35.5-/-* and wild-type siblings (Figure 1M). At 5 mM PTZ, there were small increases in activity in the diencephalon (hypothalamus) and the telencephalon (subpallium) (Figure 1N). At 10 mM PTZ, we observed increases in the hypothalamus and various regions within the rhombencephalon (Figure 1O). We also found regions that show less of an increase in activity in *cx35.5-/-* compared to wild-type within the rhombencephalon, specifically in regions that rely on the synchronous firing capabilities of Cx36 (Mauthner cells, inferior olive) (Flores et al., 2012; Yao et al., 2014; Bazzigaluppi et al., 2017). At the highest concentration (20 mM), we saw increased activity in the *cx35.5-/-* compared to wild-type in areas previously identified as associated with seizures in the telencephalon such as the pallium (Liu and Baraban, 2019) as well as the hypothalamus. These regions are similar to our findings in the wild-type animals after PTZ exposure, indicating an increase in severity of hyperactivity in these regions following treatment with PTZ in *cx35.5-/-* animals. We also observed regions that show fewer increases in activity in the rhombencephalon, relative to wild-type, similar to 10 mM PTZ, but they are less severe (Figures 1O,P). For a complete list of regional differences, please see Supplementary Table 1.

### Genetic *cx35.5* Deficiency Does Not Affect Cell Death at Baseline or After PTZ

**Figure 2:**
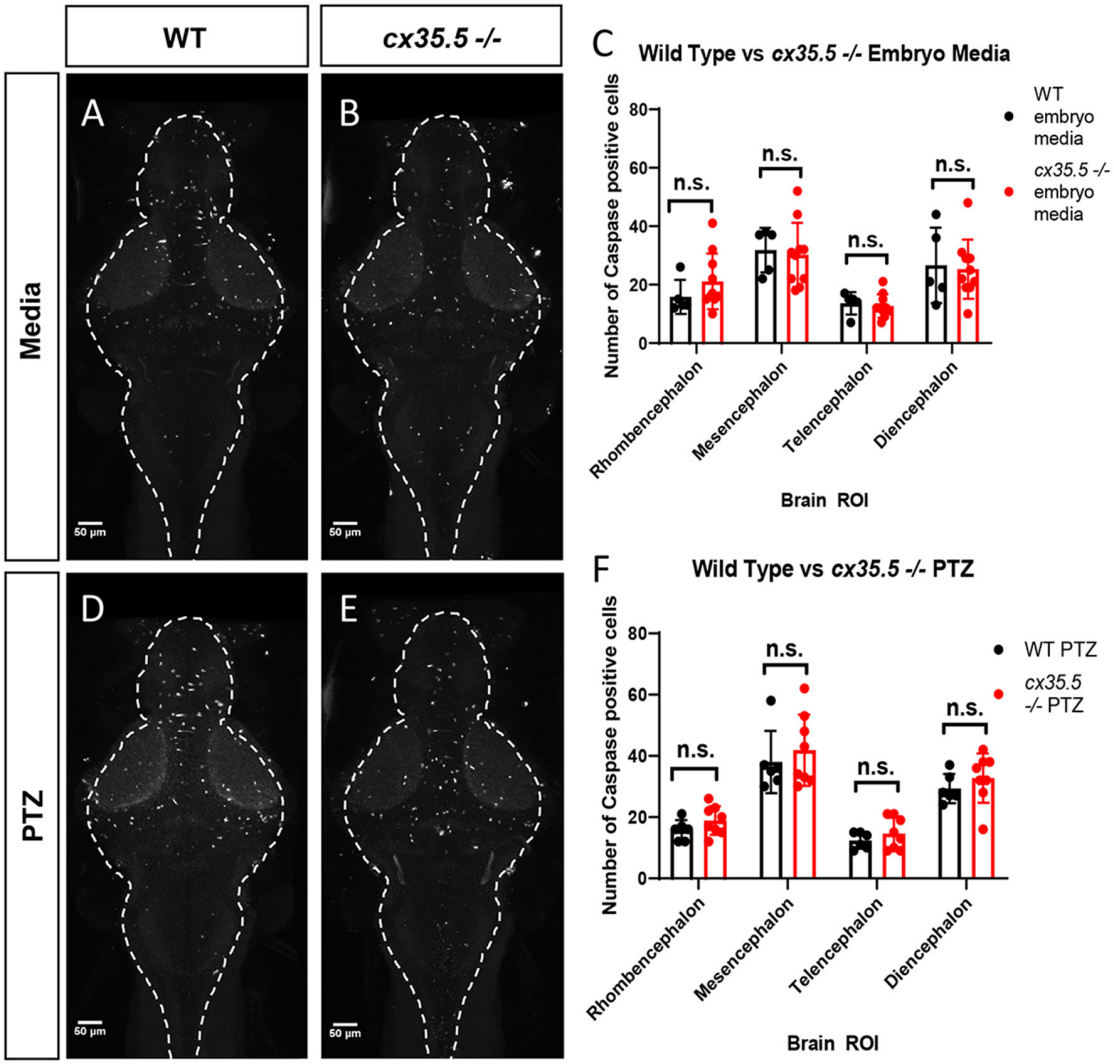
Activated caspase 3 positive cells by major brain division, comparing *cx35.5-/-* vs. wild type with and without PTZ. Representative sum stack projections of wild-type and cx35.5-/- larvae treated with media **(A, B)** or PTZ **(D, E)** and stained with anti-activated caspase-3. **(C–F)** Graphs depicting the number of activated caspase-3 positive cells in the rhombencephalon, mesencephalon, telencephalon, and diencephalon in wild-type (black) vs. *cx35.5-/-* (red) fish with treatment with **(C)** embryo medium (vehicle) or **(F)** PTZ. Data were analyzed using an unpaired *t*-test with Holm-Sidak's correction for multiple comparisons. Embryo medium (vehicle) treatment, wild type *n* = 5, *cx35.5-/- n* = 10. PTZ treatment, wild type *n* = 6, *cx35.5-/- n* = 8.

We determined that PTZ alone and PTZ in combination with *cx35.5* deficiency resulted in regional and dose-varying changes in whole-brain neuronal activity. One possible explanation is that *cx35.5* mutation may result in altered neuronal cell death, either at baseline or after PTZ, which would then alter the overall balance of brain-wide connectivity. To test this, we stained for activated caspase-3 (a marker of apoptotic cells) and quantified the number of positive cells in each of the major brain divisions (rhombencephalon, mesencephalon, telencephalon, and diencephalon, see Figure 1B for an atlas of zebrafish brain regions). We found that there were no differences at baseline (media only) in the number of activated caspase-3 positive cells between *cx35.5-/-* and wild-type siblings in any of the major brain divisions (Figures 2A–C). Additionally, no difference in the number of caspase-3 positive cells when comparing both *cx35.5-/-* and wild-type siblings after 20 mM PTZ was found (Figures 2D–F). From these data, we, therefore, conclude that changes in neuronal response in *cx35.5* animals are not likely caused by altered cell death induction.

### Creation of the Whole-Brain Cx36 Expression Map

**Figure 3:**
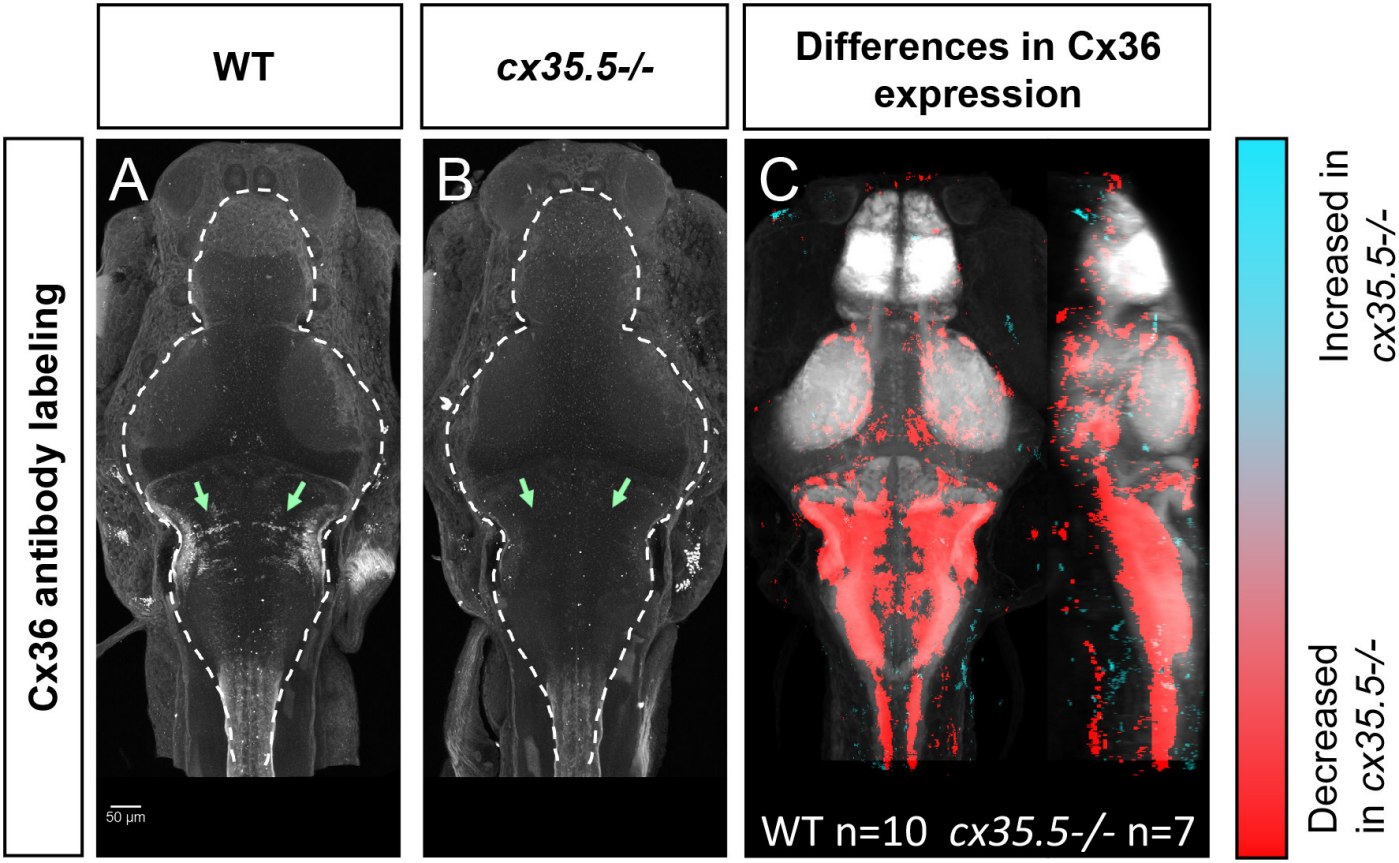
Whole-brain expression map of *cx35.5-/-* vs. wild-type zebrafish larvae immunostaining of anti-Cx36. **(A,B)** Anti-Cx36 staining in wild-type fish **(A)** and *cx35.5-/-* fish **(B)**. **(C)** Whole-brain expression map showing increased expression in *cx35.5-/-* (cyan) and increased expression in wild type (red) showing the comparison of Cx36 expression between *cx35.5*-/- and wild type. Regions with increased Cx36 expression in *cx35.5*-/- are shown in cyan and regions with decreased Cx36 expression in *cx35.5*-/- are shown in red. Wild type *n* = 10, *cx35.5-/- n* = 7.

To understand how neuronal hyperactivity affects Cx36, we created a whole-brain *expression* map to efficiently, and in a non-biased manner, measure changes in protein expression using a modified MAP-mapping processing procedure. We utilized a previously validated human anti-Cx36 antibody that recognizes all four isoforms of Cx36 in the zebrafish (*cx34.7, cx35.1, cx34.1*, and *cx35.5*). The antibody was validated against zebrafish-specific and isoform-specific generated antibodies in HeLa cells (Miller et al., 2017). Using this antibody, we stained wild-type (Figure 3A) and *cx35.5-/-* (Figure 3B) siblings. Consistent with previous studies, significant loss of anti-Cx36 staining in *cx35.5-/-* animals was detected (Figures 3A,B). To quantify Cx36 expression across the whole brain, we performed image normalization (with CMTK) and subtracted the average stack of all *cx35.5-/-* fish from each animal. We then followed the same MAP-mapping processing pipeline to quantify the Cx36/tERK ratio. tERK staining is used for morphing as a standard, consistent label (Supplementary Figure 1) and to normalize staining intensity across animals and conditions considering the sparse expression of Cx36. Statistical significance was determined through the Mann-Whitney *U* statistic, calculating statistically significant changes in the Cx36/tERK ratio for each voxel. This is represented in each of the images to follow as statistically significant increases in the Cx36/tERK ratio shown in cyan and statistically significant decreases in the Cx36/tERK ratio shown in red. FDR correction was used with *p* < 0.00005 as the cut-off for significance (Randlett et al., 2015). The resulting Cx36 expression map reveals decreases in Cx36 staining intensity in *cx35.5-/-* fish compared to wild-type siblings in regions such as the mesencephalon (optic tectum), rhombencephalon (rhombomeres, mauthner cells), etc. (Figure 3C). See Supplementary Table 2 for a complete list of regional changes. We then applied this same method to examine Cx36 expression after PTZ.

### Reduced Cx36 Expression Following PTZ Exposure

**Figure 4:**
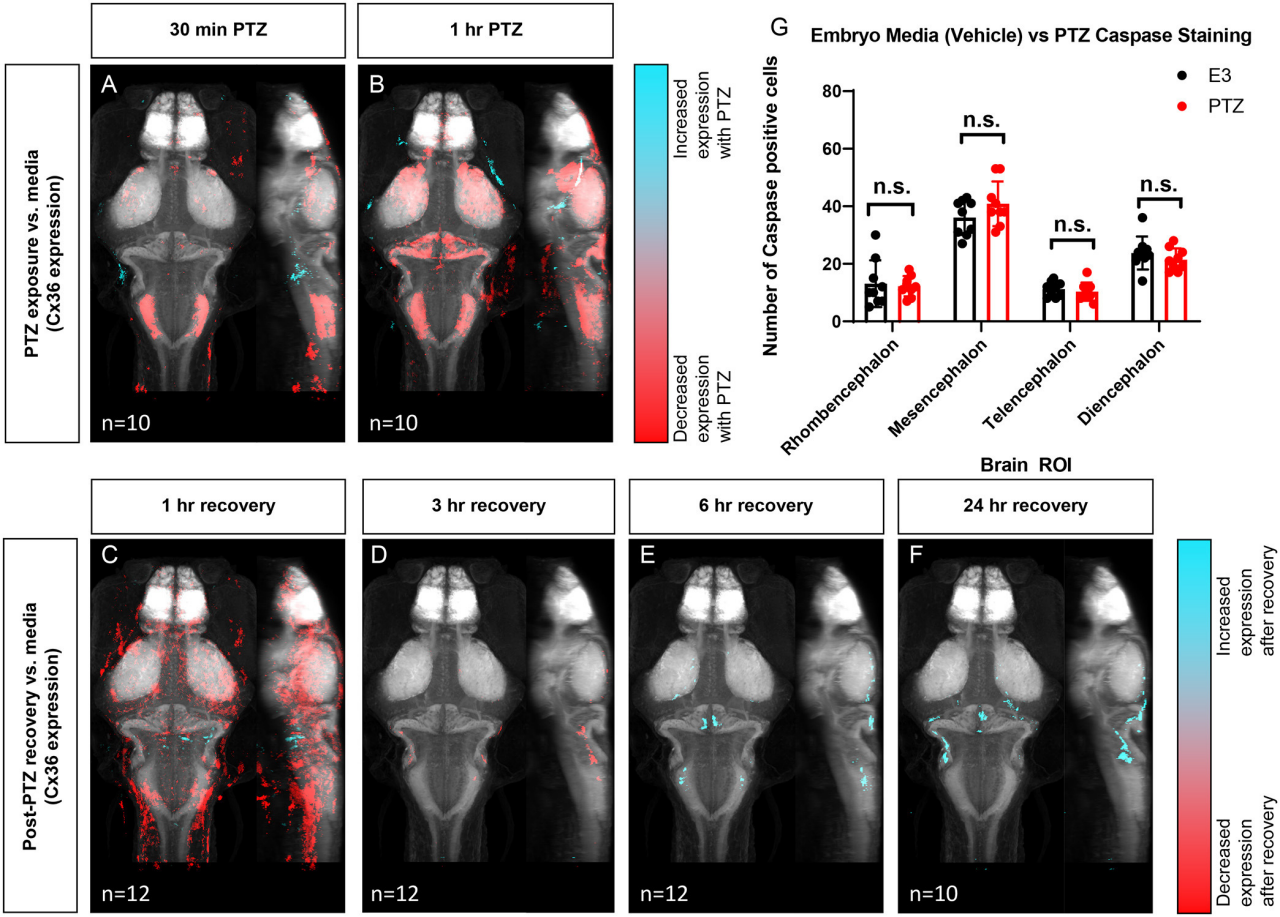
Wild type whole-brain immunostaining Cx36 expression map in E3 vs. PTZ treated zebrafish larvae. Dorsal and lateral view of zebrafish larvae brain. Whole-brain expression of Cx36 using an anti-Cx36 antibody and tERK. Cyan indicates increases in Cx36 labeling in PTZ treated fish, and red indicated decreases in Cx36 labeling in PTZ treated fish. **(A)** After 30 min of 20 mM PTZ exposure (*n* = 10). **(B)** After 1 h of 20 mM PTZ exposure (*n* = 10). **(C)** 1-h recovery after PTZ is removed, *n* = 12. **(D)** 3 h of recovery after PTZ is removed, *n* = 12. **(E)** 6 h of recovery after PTZ is removed, *n* = 12. **(F)** 24 h of recovery after PTZ is removed, *n* = 10. **(G)** A graph depicting the number of activated caspase-3 positive cells in the rhombencephalon, mesencephalon, telencephalon, and diencephalon in wild-type fish with treatment with embryo medium (vehicle) (Black) or PTZ (Red). Data were analyzed using an unpaired *t*-test with Holm-Sidak's correction for multiple comparisons, *n* = 9.

Next, to determine if exposure to PTZ changes Cx36 expression, we compared the Cx36 expression map between PTZ-treated animals and untreated (media only) animals. Due to the time-frame of Cx36 turnover (half-life of ~1–3 h) (Flores et al., 2012), we did not expect to see changes in expression after only 15 min of PTZ exposure. As such, we exposed fish to PTZ for both 30 min and 1 h to ensure we captured changes in expression. After 30 minutes of PTZ exposure, we found a global decrease in Cx36 labeling (Figure 4A). A similar but more pronounced effect was observed after 1 h (Figure 4B). We saw decreases in Cx36 expression in the mesencephalon (optic tectum), the diencephalon (retinal arborization fields), and in the rhombencephalon in rhombomere 7, an area that is important for motor behavior (Figures 4A,B), see Figure 1 for an atlas of zebrafish brain regions. After 1 h of PTZ, there was also a decrease in expression within the cerebellum (Figure 4B), an area that relies heavily on Cx36 for synchronous firing. For a complete list of ROIs with changes, see Supplementary Table 2. Together, these data reveal that Cx36 expression is reduced following exposure to PTZ after 30 min, and this is exacerbated after 1 h of exposure.

### Recovery of Cx36 Expression Following Cessation of PTZ Exposure

To test whether Cx36 expression recovers after the removal of PTZ, we created Cx36 expression maps for animals exposed to 20 mM PTZ for 1 h and then allowed them to recover in embryo media for 1, 3, 6, or 24 h after PTZ removal. Compared to animals not exposed to PTZ (media only), Cx36 expression was still significantly decreased in the telencephalon (pallium, subpallium) and diencephalon (habenula, pretectum), after 1 h of recovery, but there were some increases in expression in restricted areas in the rhombencephalon (Figure 4C). The decrease in Cx36 expression was almost entirely recovered after 3 h (Figure 4D). Interestingly, expression is then slightly increased by 6 h of recovery in the mesencephalon (optic tectum neuropil), and the cerebellum (Figure 4E). This is maintained 24 h later (Figure 4F). For a complete list of regions that show changes in expression, see Supplementary Table 2. These alterations in expression were not due to cell death resulting from long-term PTZ exposure as no significant differences in the number of caspase-3 positive cells in between untreated (media only) vs. those treated with 20 mM PTZ for 1 h (Figure 4G) we detected.

### Acute Blockade of Cx36 Increases Neuronal Hyperactivity Following PTZ Exposure

**Figure 5:**
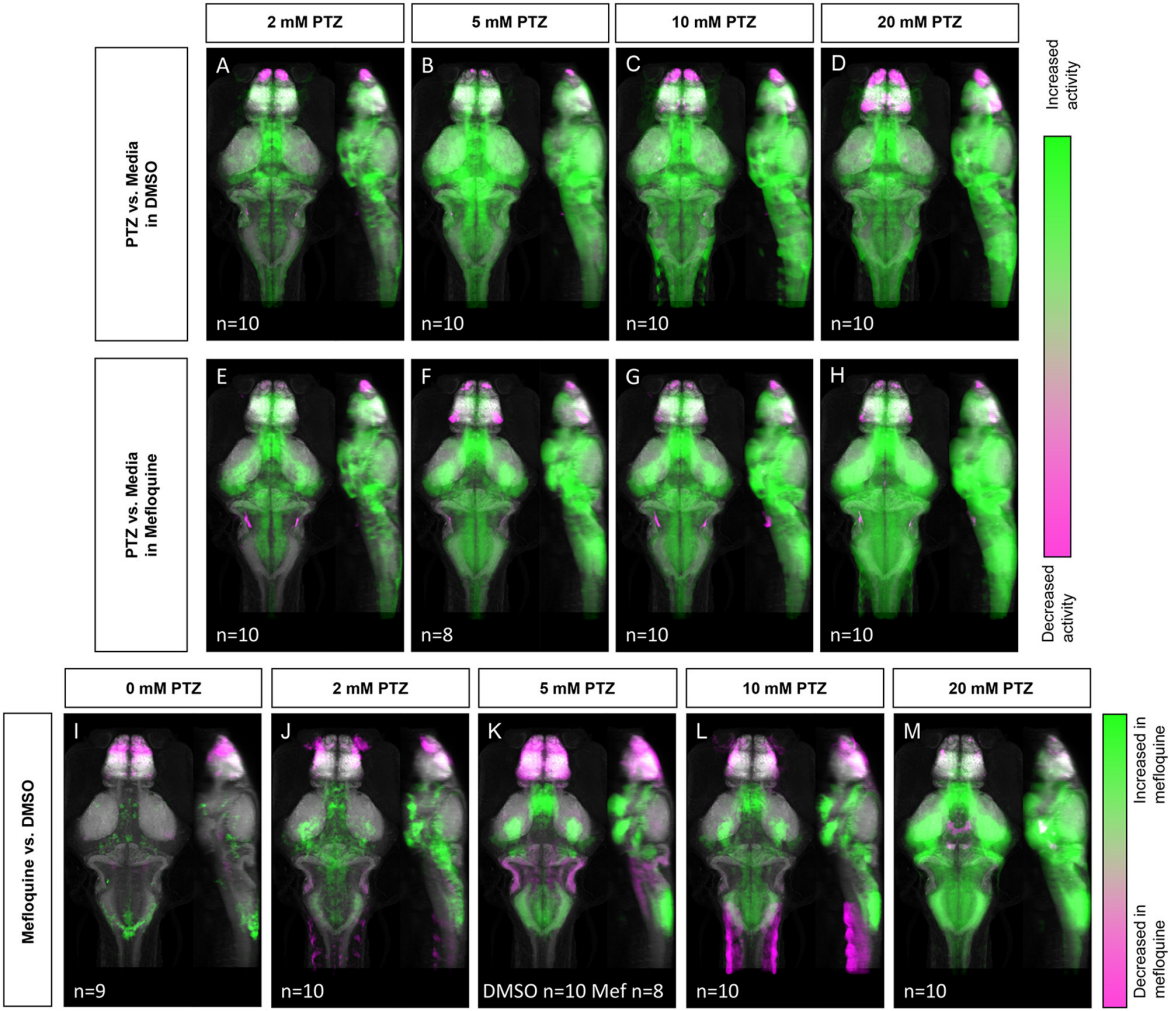
Whole-brain activity map showing significant regional differences following Cx36 blocking drug mefloquine and PTZ exposure in wild-type zebrafish larvae. Dorsal and lateral view of zebrafish larvae brain. **(A–D)** Images show regions with significantly increased (green) or decreased (magenta) activity compared to DMSO and embryo medium (vehicle) treated fish (*n* = 9). **(A)** 2 mM PTZ (*n* = 10). **(B)** 5 mM PTZ (*n* = 10) **(C)** 10 mM PTZ (*n* = 10). **(D)** 20 mM PTZ (*n* = 10). **(E–H)** Images show regions with significantly increased (green) or decreased (magenta) activity compared to mefloquine and embryo medium (vehicle) treated fish (*n* = 9). **(E)** 2 mM PTZ (*n* = 10). **(F)** 5 mM PTZ (*n* = 8). **(G)** 10 mM PTZ (*n* = 10). **(H)** 20 mM PTZ (*n* = 10). **(I–M)** Images show regions with significantly increased (green) or decreased (magenta) activity in mefloquine treated fish, compared to DMSO (vehicle) treated fish. **(I)** embryo medium (0 mM PTZ) (*n* = 9). **(J)** 2mM PTZ (*n* = 10). **(K)** 5 mM PTZ (DMSO *n* = 10, mefloquine *n* = 8) **(L)** 10 mM PTZ (*n* = 10). **(M)** 20 mM PTZ (*n* = 10). Mef: mefloquine.

Given that PTZ-induced neuronal hyperactivity resulted in decreased Cx36 expression, we next tested whether the acute reduction of Cx36 function may contribute to further susceptibility to neuronal hyperactivation, i.e., whether PTZ-induced Cx36 reduction is maladaptive. To acutely inhibit Cx36 function, we utilized a Cx36-specific blocking drug, mefloquine, and examined changes in neuronal activity (Harris and Locke, 2008). The effects of mefloquine were assessed by comparing the activity maps of wild-type fish treated with DMSO (vehicle) or 25 μM mefloquine for 3 h before exposure to varying concentrations of PTZ (0–20 mM). Similar to our wild-type activity mapping (Figures 1D–G), we observed broad increases in neuronal activity in DMSO treated animals following exposure to PTZ in a dose-dependent manner (Figures 5A–D). These increases were greater compared to wild-type animals not exposed to DMSO (Figures 1D–G), likely due to the lower neural activity baseline caused by the mild inhibitory effects of DMSO on excitatory currents (Supplementary Figure 2, Supplementary Table 5) (Lu and Mattson, 2001; Tamagnini et al., 2014). At 2–5 mM PTZ, we saw increases in activity in the mesencephalon (optic tectum, neuropil), rhombencephalon (cerebellum), telencephalon (pallium), and diencephalon (hypothalamus) (Figures 5A,B, see Figures 1B,C for referenced zebrafish brain regions). There were also decreases in activity in the olfactory bulb (Figures 5A,B). At 10 mM we observed increases in similar regions, with greater increases seen in the hypothalamus, decreases in the olfactory bulb, and small decreases in the hypothalamus and pallium (Figure 5C). Finally, at 20 mM PTZ increases in neuronal activity in similar regions as the previous doses were observed, with the greatest increases seen in the hypothalamus. Decreases in the olfactory system, hypothalamus, and pallium (Figure 5D) were also observed. In fish treated with mefloquine, we found very similar overall patterns as the DMSO treated fish (Figures 5A–D), with progressively higher neuronal activity with increasing PTZ concentrations (Figures 5E–H).

Next, we compared mefloquine vs. DMSO treated siblings at different concentrations of PTZ. In the absence of PTZ, the mefloquine treated fish showed increases and decreases in neuronal activity in different brain regions, compared to DMSO treated siblings (Figure 5I). Specifically, we saw moderate increases in the hypothalamus, cerebellum, and mesencephalon (tegmentum). There were decreases in neuronal activity compared to DMSO treated controls in the telencephalon, specifically in the olfactory bulb and subpallium (Figure 5I). At 2 mM PTZ, mefloquine treated fish showed increases in the major regions associated with PTZ exposure (Figure 1D), compared to DMSO-treated fish. Increases in the hypothalamus, diencephalon (retinal arborization fields, pretectum), and telencephalon (subpallium) were found. There were decreases in the olfactory bulb and other regions of the telencephalon compared to controls (Figure 5J). At 5 mM PTZ, we found similar regions of increased activity in mefloquine treated fish, but we also saw regions that showed decreased activity compared to control within both the telencephalon and the rhombencephalon (Figure 5K). Some rhombencephalon regions with decreased activity are known to rely on Cx36 functionality, specifically the inferior olive and Mauthner cells (Flores et al., 2012; Yao et al., 2014; Bazzigaluppi et al., 2017). At 10 and 20 mM PTZ, we observed similar increases in activity in mefloquine treated fish, each increasing with PTZ dose, and a decrease in activity compared to control in the telencephalon, which was less severe than 5 mM, in these two groups (Figures 5L,M). At 20 mM we observed a decrease in activity in the hypothalamus and mesencephalon (oculomotor nuclei) compared to wild-type, which was not observed at other doses (Figure 5M). For a complete list of regions changed, see Supplementary Table 3. Compared to *cx35.5-/-* animals (Figures 1L–P), the activity increases we found in the mefloquine-treated animals were more wide-spread. Overall, these results indicate that the acute reduction of Cx36 functionality results in increased susceptibility to PTZ-induced neuronal hyperactivity.

### Reduced Mefloquine-Induced PTZ Susceptibility in *cx35.5* Mutants

**Figure 6:**
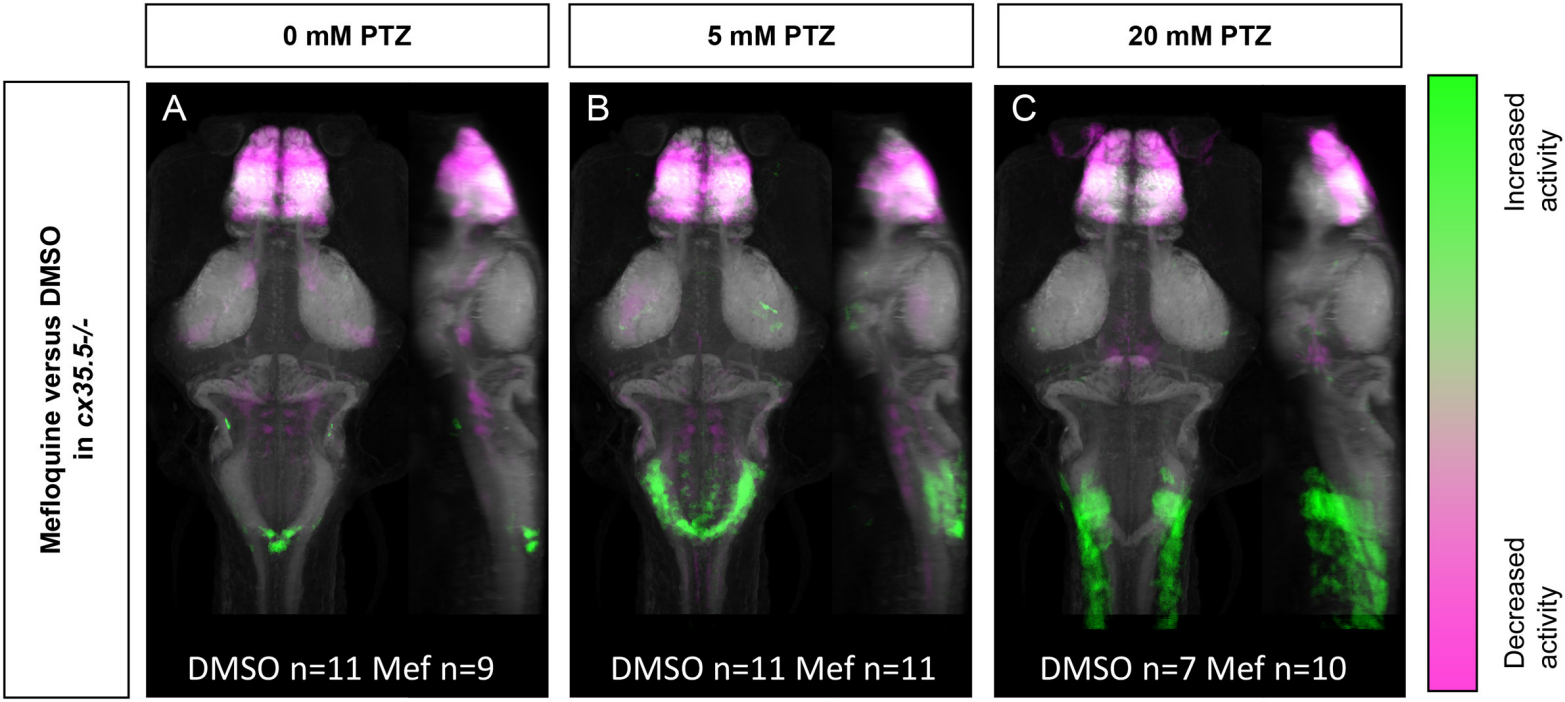
Whole-brain activity of *cx35.5-/-* animals treated with mefloquine vs. DMSO. Dorsal and lateral views of zebrafish larvae brain are shown. Images show regions with significantly increased (green) or decreased (magenta) activity in *cx35.5-/-* animals treated with mefloquine compared to *cx35.5-/-* animals treated with DMSO. **(A)** Embryo medium (0 mM PTZ) (*cx35.5-/-* mefloquine *n* = 9, *cx35.5-/-* DMSO *n* = 11). **(B)** 5 mM PTZ (*cx35.5-/-* mefloquine *n* = 11, *cx35.5-/-* DMSO *n* = 11). **(C)** 20 mM PTZ (cx35.5-/- mefloquine *n* = 10, *cx35.5-/-* DMSO *n* = 7). Mef: mefloquine.

The more severe mefloquine phenotype, compared to *c*x35.5 mutants, may be caused by acute inhibition of both Cx35.5 and other Cx36 isoforms. Mefloquine exerts various off-target effects at moderate concentrations. Additionally, the *cx35.5* mutants still have low levels of Cx36 labeling (Miller et al., 2017, and Figure 3) meaning that the model is not a complete knockout. As such, we wanted to determine what changes in neuronal activity after treatment with mefloquine are due to these various off-target effects. To answer this question, we examined the effects of mefloquine on *cx35.5-/-* fish, with and without PTZ. We compared the differences in neuronal activity in mefloquine treated and DMSO treated *cx35.5-/-* fish, with no PTZ (embryo media only), 5 mM PTZ, or 20 mM PTZ (Figure 6). In all conditions, we observed decreases in neuronal activity within the telencephalon (olfactory bulb, subpallium, pallium), the diencephalon (habenula, retinal arborization fields), and the rhombencephalon (inferior olive). In both the embryo media and 5 mM PTZ conditions, we observed increases in neuronal activity following the administration of mefloquine in a small region of the rhombencephalon (area postrema, neuropil, rhombomere 7). At 20 mM PTZ, we observe small increases in activity in smaller neuron clusters and observed significant decreases in the telencephalon (olfactory bulb, pallium, subpallium) diencephalon (habenula), and rhombencephalon (Figure 6C). These changes in neuronal activity are *cx35.5*-independent and are likely off-target effects and may be due to inhibition of other Cx36 isoforms. Nevertheless, the effects of mefloquine are greatly reduced in *cx35.5-/-* animals, compared to its effects on wild-type animals (compare Figures 5A–D to Figures 6A–C). Specifically, the pronounced increase in neuronal activity in the hypothalamus, pre-tectum, and subpallium regions was not seen in the *cx35.5-/-* animals. This result indicates that a large portion of the effects of mefloquine are dependent on Cx36 expression. For a complete list of regions changed, see Supplementary Table 4.

## Discussion

The goal of this study was to understand the reciprocal relationship between Cx36 and neuronal hyperactivity on a brain-wide scale. We utilized MAP-mapping to quantify neuronal activity and protein expression across the whole-brain. Through this, we characterized the complex nature of this relationship and its dependence on many factors including brain region, drug dose, and exposure time. We found that chronic deficiency in the *cx35.5* mutants altered susceptibility to PTZ-induced neuronal hyperactivity in a region-specific manner. We developed a whole-brain quantification method for the expression of zebrafish Cx36 isoforms and found that PTZ exposure resulted in an acute decrease in Cx36 protein, which recovered and slightly overshot when PTZ was removed. Finally, we observed that acute inhibition Cx36 by mefloquine resulted in a broad increase in the susceptibility to PTZ induced hyperactivity. Taken together, these results suggest that Cx36 acts to prevent hyperactivity within the brain, and that loss of Cx36 protein, both acute (potentially due to previous hyperactivity) and chronic, results in an increase in susceptibility to hyperactivity.

### PTZ Exerts Brain-Wide and Region-Specific Effects

PTZ has been used as a chemical convulsant in many animal models of epilepsy and is known to cause generalized but non-uniform brain activation (Shehab et al., 1992; Nehlig, 1998; Baraban et al., 2005; Szyndler et al., 2009; Baxendale et al., 2012; Diaz Verdugo et al., 2019; Yang et al., 2019). Previous zebrafish calcium imaging studies have found increases in neuronal activity and synchronicity after PTZ administration, with differential recruitment of different brain regions. For example, Liu and Baraban found that increases in neuronal activity originate in the pallium and then propagate to the hindbrain (Liu and Baraban, 2019). Additionally, it was found that there were significant increases in neuronal connectivity in each of the regions observed (Diaz Verdugo et al., 2019). Studies in younger zebrafish (2 dpf) also found changes in *c-fos* expression following the administration of PTZ in a manner similar to our MAP-map (Baxendale et al., 2012). We were able to generate activity maps for different PTZ concentrations that identify the same regions described before (pallium, optic tectum, hindbrain), but also additional brain regions that have not been implicated previously (e.g., the hypothalamus). This demonstrates the importance of identifying brain-wide region-specific effects when examining hyperactivity. Taken together, these results illustrate the unique dose-varying whole-brain effects of PTZ that can be expanded upon in future work.

### *cx35.5* Knockdown Causes Region-Specific Changes in Hyperactivity Following PTZ Administration

In addition to characterizing the effect of PTZ on whole-brain activity in wild-type animals, we gained insight into PTZ's effects in *cx35.5-/-* zebrafish. We saw increases in regions identified in our PTZ dose-response experiment, indicating more severe increases in neuronal hyperactivity following the administration of PTZ in those regions (Figure 1). These results are consistent with previous behavior that showed increased susceptibility of Cx36 mutant to PTZ-induced severe seizure-like behaviors than their wild-type counterparts (Jacobson et al., 2010). In addition to activity increases, we observed significant decreases in neuronal activity at 10 mM PTZ concentrations. These decreases were observed in the rhombencephalon, specifically in regions that rely on Cx36 for synchronous firing (inferior olive, Mauthner cells) (Flores et al., 2012; Yao et al., 2014; Bazzigaluppi et al., 2017). These results are important, as it is the first study to show regional differences in neuronal activity between Cx36-deficient and wild-type animals, which indicates the lack of generalizability from region to region within the brain when examining connexin proteins.

### PTZ-Induced Hyperactivity Causes a Regionally-Specific Decrease in Cx36 Expression

To further understand the relationship between Cx36 and hyperactivity, we asked the reciprocal question: how does hyperactivity affect Cx36? Similar to the seizure susceptibility studies, work to identify this relationship has remained conflicting (Söhl et al., 2000; Laura et al., 2015; Motaghi et al., 2017; Wu et al., 2017). Previous approaches used to address this question (e.g., qPCR, western blot) lacked the necessary spatial resolution to determine if the effects of hyperactivity on Cx36 vary based on the brain region. To address these shortcomings, we developed a novel method for quantifying the whole-brain expression of the Cx36 protein, using antibody staining in conjunction with a modified MAP-mapping technique (Figure 3). We were, therefore, able to determine that there are regional and exposure time differences in the reduction of Cx36 in response to seizure induction using PTZ. Specifically, we saw reductions in a region-specific manner after exposure to PTZ for 30 min, and those reductions were greater after 1 h of PTZ exposure (Figure 4). Therefore, we have determined PTZ exerts region-specific effects on Cx36 and that changes found in one region of the brain may not be directly applicable to other regions.

### Reduction in Cx36 Expression Following Hyperactivity Is Acute and Recovers Over Time

After observing a decrease in Cx36 expression following exposure to PTZ, we measured the temporal patterns of this change. We found that the change in Cx36 expression was acute: it occurred within the 1st h of PTZ exposure and was almost fully recovered by 3 h (Figures 4C,D). The recovery was then slightly overshot. Cx36 was overexpressed in the optic tectum and cerebellum as well as other brain regions, and this overexpression was maintained 24 h later (Figures 4E,F). Because the reduction was not caused by an increase in cell death (Figure 4G), this effect is likely due to an increase in endocytosis and degradation of the Cx36 protein. Various studies have shown that activity-dependent modulation of Cx36 proteins exists (Smith and Pereda, 2003; Haas et al., 2016) and endocytosis is a likely mechanism by which this can occur (Flores et al., 2012).

### Acute Reduction in Cx36 Functionality Leaves Organisms More Susceptible to PTZ Induced Hyperactivity

To solidify the relationship between hyperactivity and Cx36, we studied how acute blockade of Cx36 affects susceptibility to hyperactivity. Is the reduction in Cx36 after PTZ exposure adaptive, maladaptive, or inconsequential? To answer this question, we utilized the Cx36-specific blocking drug mefloquine and expose mefloquine treated and untreated fish to PTZ to observe differences. Mefloquine is an anti-malarial drug that selectively blocks Cx36 and Cx50. Previous studies utilized quinine which has more off-target effects. It is hypothesized that mefloquine blocks Cx36 by binding to the inside of the pore, preventing the flow of ions through that pore (Harris and Locke, 2008). We found a significant increase in neuronal hyperactivity following treatment with PTZ in the mefloquine treated fish compared to control (Figure 5). This result indicates a reduction in Cx36 in all cases (acute and chronic) is detrimental and leads to an altered severity of hyperactivity. This result is consistent with Voss et al. (2009), who found using different gap junction blockers, animals exhibited increases in neuronal activity in the cerebral cortex, but inconsistent with Franco-Pérez et al. (2015) which found a decrease in seizure-like motor behavior and activity within the motor cortex. This may be due to differences in output measures. Each of these studies examined a different brain region and as we have shown, Cx36 exerts region-specific effects. Zebrafish do not have a motor cortex, therefore we are unable to confirm or reject the results found by Franco-Pérez et al. (2015), however, in our model, we do observe increases in activity within the pallium (analogous to the cortex in mammals).

At moderate doses (6–25 μM), mefloquine can exhibit off-target effects of varying degrees (McArdle et al., 2006; Caridha et al., 2008; Harris and Locke, 2008). To better understand the non-Cx36 effects of mefloquine, we treated *cx35.5-/-* fish with mefloquine and quantified changes in neuronal activity both at rest (in embryo medium) and after PTZ (5 mM). We observed major decreases in neuronal activity within the forebrain and a slight decrease in the rhombencephalon in both conditions. Additionally, we observed a slight increase in neuronal activity in the rhombencephalon, which was exacerbated slightly by PTZ (Figure 6). We attribute these effects to the off-target effects of mefloquine. As such, in wild-type animals, changes we observed in PTZ sensitivity (Figures 5J–M) in other regions are more likely to be caused by Cx36 blockade.

We found that the effects of mefloquine on PTZ-induced neuronal hyperactivity (Figure 5) were greater than that of *cx35.5* knockout (Figure 1). This may be due to the difference between acute (mefloquine) vs. congenital (*cx35.5*) perturbation in overall Cx36 function. In the *cx35.5* mutants, there may be compensatory mechanisms that can partially ameliorate the effects of reduced Cx36 function (Rossi et al., 2015). Additionally, it is important to note that while overall Cx36 levels (as measured by Cx36 immunolabeling) is significantly reduced in *cx35.5-/-* animals, there is still some residual Cx34.1 expression. Thus, the more severe phenotype seen in the mefloquine treated animals may reflect a more complete inhibition of all Cx36 isoforms. Finally, while we selected mefloquine due to its selective activity toward Cx36, it does still exert off-target effects, including blockage of other connexin proteins and may be toxic (Cruikshank et al., 2004). Nevertheless, the main effect of mefloquine on PTZ-induced hyperactivity depends on Cx35.5 expression (Figure 6), which supports our interpretation that acute knockdown of zebrafish Cx36 proteins by mefloquine results in increased susceptibility to neuronal hyperactivity (Figure 5).

### Cx36 as a Contributing Factor Regulating the Brain's Response to Hyperactivity

A plausible relationship to human disease is in Juvenile Myoclonic Epilepsy (JME). Individuals with JME have a higher likelihood of harboring a specific intronic SNP in the *Cx36* gene (Mas et al., 2004; Hempelmann et al., 2006). This SNP has been hypothesized to affect splicing enhancers of the gene, therefore affecting the translation of the protein (Mas et al., 2004). While Cx36 is not the only cause for diseases like JME, it may be a contributing factor. Based on our results, loss of Cx36 would be predicted to make an individual more susceptible to other factors leading to hyperactivity, increasing the severity of hyperactivity (Figures 1, 5). This is particularly relevant as Cx36 expression is highest during development and decreases over time (Belousov and Fontes, 2013) and JME first appears in children and adolescents.

Our work demonstrates that Cx36 appears to reduce PTZ induced hyperactivity in specific brain regions and that loss of the protein is detrimental to that process. We were able to determine where in the brain we see effects in addition to when those changes occur. This work provides a basis for a better understanding of the role of Cx36 and PTZ induced hyperactivity.

## Data Availability Statement

The raw data supporting the conclusions of this article will be made available by the authors, without undue reservation.

## Ethics Statement

The animal study was reviewed and approved by Virginia Tech Institutional Animal Care and Use Committee.

## Author Contributions

AB, MM, and YP conceived the study. AB, KC, MM, and YP designed the experiments. AB performed the experiments. AB, IW, and YP analyzed the data. KC and MM contributed to the MAP-mapping analysis. AB and YP wrote the manuscript. All authors contributed to editing the article and approved the submitted version.

## Conflict of Interest

The authors declare that the research was conducted in the absence of any commercial or financial relationships that could be construed as a potential conflict of interest.

## Supplementary Material

Supplementary Figure 1 Comparison of tERK staining intensity. Graph depicting mean tERK staining intensity between all Cx36 co-stained groups. There is no significant differences between media and PTZ treated groups for each exposure and recovery condition.

Supplementary Figure 2 Whole-brain activity map showing effects of DMSO in wild-type animals. Dorsal and lateral view of zebrafish larvae brain. Images show regions with increased (green) and decreased (magenta) activity in DMSO treated wild-type animals versus embryo media (no DMSO) treated wild-type animals (DMSO *n* = 9; no DMSO *n* = 10).
