# Supplementary material for: Effects of Constitutive and Acute Connexin 36 Deficiency on Brain-Wide Susceptibility to PTZ-Induced Neuronal Hyperactivity
Source: Front Mol Neurosci. 2021 Jan 11;13:587978. doi: 10.3389/fnmol.2020.587978 (PMC7829467; doi:10.3389/fnmol.2020.587978)
Supplement: Supplementary Figure 1 — Comparison of tERK staining intensity. Graph depicting mean tERK staining intensity between all Cx36 co-stained groups. There is no significant differences between media and PTZ treated groups for each exposure and recovery condition. [file Image_1.pdf]

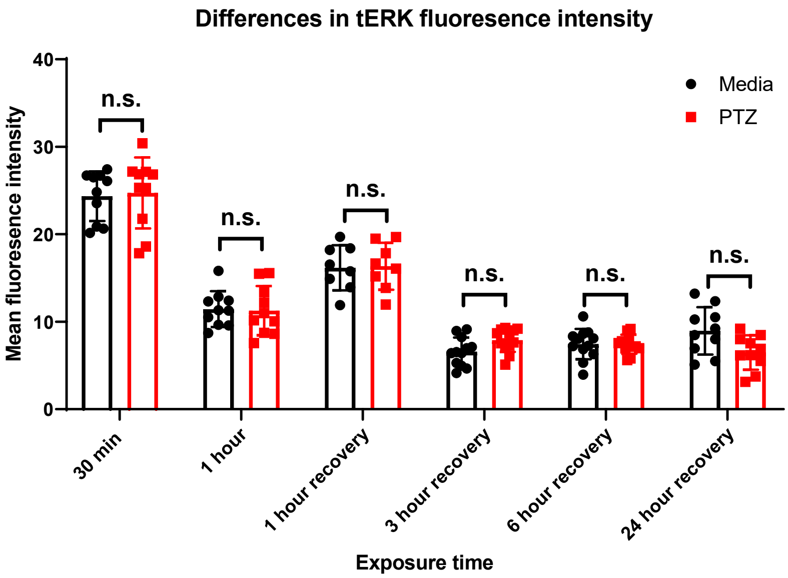

Supplementary Figure 1. Comparison of tERK staining intensity. Graph depicting mean tERK staining intensity between all Cx36 co-stained groups. There is no significant differences between media and PTZ treated groups for each exposure and recovery condition.

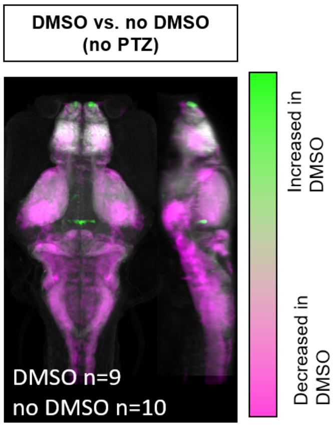

Supplementary Figure 2. Whole-brain activity map showing effects of DMSO in wild-type animals. Dorsal and lateral view of zebrafish larvae brain. Images show regions with increased (green) and decreased (magenta) activity in DMSO treated wild-type animals versus embryo media (no DMSO) treated wild-type animals (DMSO n=9; no DMSO n=10).
